# Case report: A rare case of imiquimod-induced atypical pemphigus vulgaris

**DOI:** 10.3389/fmed.2022.1054544

**Published:** 2022-11-24

**Authors:** Francesco Moro, Davide Ciccone, Luca Fania, Feliciana Mariotti, Adele Salemme, Siavash Rahimi, Sabatino Pallotta, Giovanni Di Zenzo

**Affiliations:** ^1^Dermatology Clinic, Istituto Dermopatico dell’Immacolata (IDI)-IRCCS, Rome, Italy; ^2^Molecular and Cell Biology Laboratory, Istituto Dermopatico dell’Immacolata (IDI)-IRCCS, Rome, Italy; ^3^Department of Anatomic Pathology, Istituto Dermopatico dell’Immacolata (IDI)-IRCCS, Rome, Italy

**Keywords:** pemphigus vulgaris, imiquimod, adverse events, Dsg1, Dsg3, non-desmoglein antigen

## Abstract

**Background:**

Pemphigus vulgaris is an autoimmune intraepithelial bullous disease involving the skin and the mucous membranes. Imiquimod, a topical therapy for skin basal cell carcinoma, is an amine that induces the production of tumor necrosis factor alfa, interleukin-1 and other cytokines. Pemphigus induced by drugs has been frequently reported, mostly after systemic therapy.

**Case presentation:**

We present the case of a 50-year-old man who developed skin, intraoral, and genital mucosae lesions 3 days after a treatment with Imiquimod for multiple superficial basal cell carcinoma of the trunk. Direct and indirect immunofluorescence results were compatible with the diagnosis of pemphigus vulgaris. Enzyme-linked immunosorbent assay was negative for desmoglein 1 and 3, but interestingly, by immunoblotting on keratinocyte extracts a band of 170 kDa was obtained by IgG. The patient, after interrupting Imiquimod application, started a treatment with prednisolone and in 4 weeks showed a complete remission.

**Conclusion:**

Topical Imiquimod therapy might induce atypical pemphigus vulgaris in some patients.

## Introduction

Pemphigus vulgaris (PV) is an autoimmune intraepithelial bullous disease involving the skin and the mucous membranes clinically characterized by erosions and flaccid bullae. PV affects patients with a male-to-female ratio of 1:1.8 (age range 50–60 years), with an annual incidence of 2–10 per one million inhabitants in central Europe and 4.2 in the American general population, but it depends on the geographical area and the ethnicity (1, 2). PV is caused by anti-desmoglein 1 (Dsg1) and/or anti-desmoglein 3 (Dsg3) autoantibodies responsible for the loss of adhesion between keratinocytes leading to acantholysis and intra-epidermal blisters (1). However, several other non-desmoglein autoantibodies have been linked to acantholysis observed in PV and these cases are usually termed atypical pemphigus (2).

Imiquimod (Aldara; 3M Pharmaceuticals, St. Paul, MN, USA) is an imidazoquinolone amine that acts as a ligand of the Toll-like receptor 7 inducing the production of tumor necrosis factor (TNF)-alfa, interleukin (IL)-1, IL-1 antagonists, IL-6, IL-10, IL-12, and additional cytokines including interferon (IFN)-alfa (3).

Imiquimod is used as topical therapy to treat basal cell carcinoma (BCC) with a high histological clearance and good cosmetic outcomes (4).

Pemphigus induced by drugs has rarely been described with pemphigus foliaceus as the most common variant (5). A great variety of drugs have been implicated in the mechanism of acantholysis such as allopurinol and angiotensin-converting-enzyme inhibitors. In most cases the drug implicated was used for systemic therapy (5).

## Case description

**FIGURE 1:**
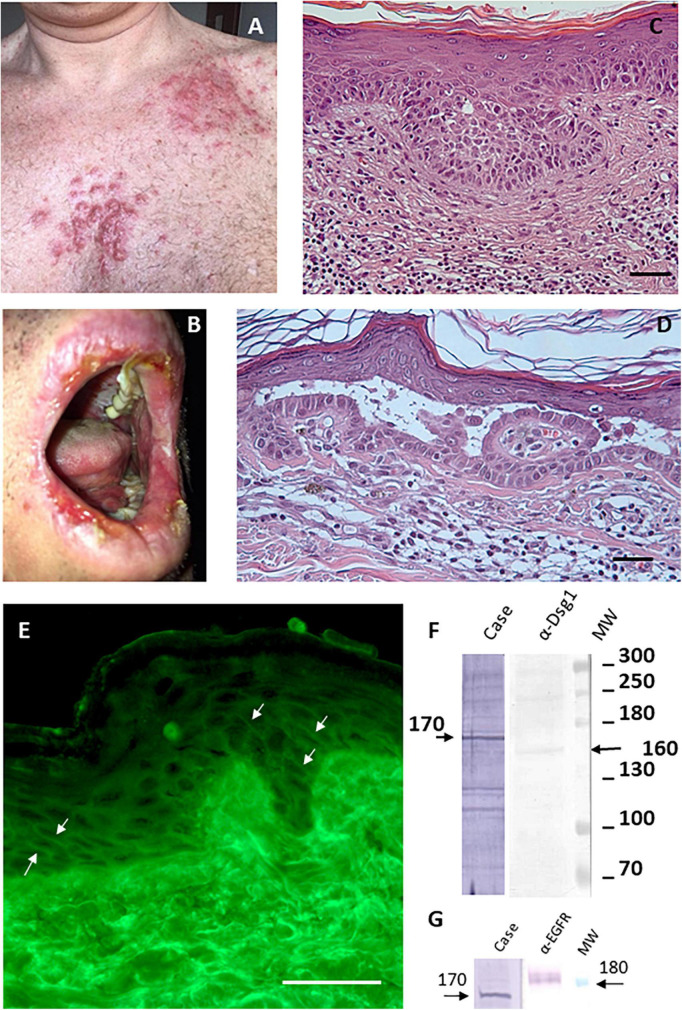
Imiquimod-induced pemphigus vulgaris. **(A)** Papules and nodules on erythematous base in seborrheic distribution. **(B)** Painful erosions on the lips. **(C)** Diagnosis of superficial variant of basal cell carcinoma made through histological analysis (scale bar: 100 μm). **(D)** Histological examination shows intraepidermal acantholytic blister with a suprabasal cleavage plane compatible with atypical pemphigus vulgaris (scale bar: 100 μm). **(E)** Direct immunofluorescence displayed a weak intercellular deposition of IgG (scale bar: 100 μm). **(F)** By IB on keratinocyte extracts a band of 170 kDa was obtained by IgG (case), the molecular weight marker and anti-Dsg1 control antibody confirm that reactivity involves an unknown antigen of 170 kDa. **(G)** IB on keratinocyte extracts shows that the 170 kDa band does not correspond to EGFR detected by a specific α-EGFR polyclonal antibody.

A 50-year-old Caucasian man presented in January 2017 with papules, nodules and erosive lesions on the skin of the upper trunk, on the lips, oral cavity, pharynx, larynx, and on the genital mucosae. On clinical examination, the skin showed papules, nodules on erythematous base in a seborrheic distribution (Figure 1A). The erosions on the lips and mucous membrane lesions were painful (Figure 1B). The Nikolsky’s sign in our patient was positive. At physical examination no other remarkable sign was detected. The patient suffers from hypertension and a diagnosis of multiple superficial BCCs was set 2 months before. In his family no one else had similar cutaneous symptoms and there is no history of autoimmune diseases.

**FIGURE 2:**
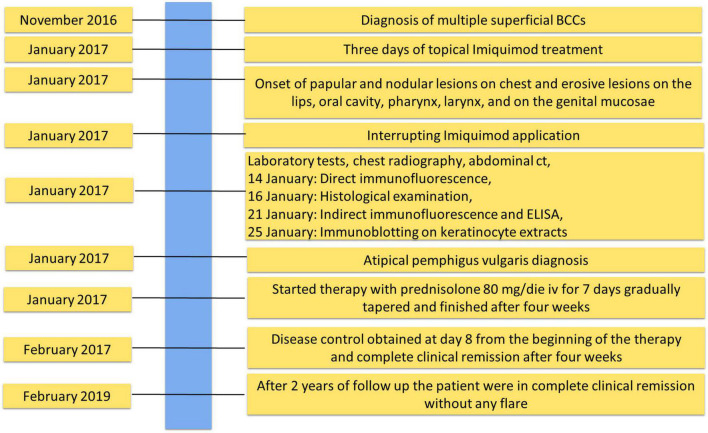
Timeline with relevant data about onset, diagnosis, and therapy of atypical pemphigus vulgaris.

The patient was being treated with Imiquimod for multiple superficial BCCs for 3 days before the onset of the lesions (Figure 1C). The findings of routine laboratory tests, chest radiography and abdominal computed tomography scan were within normal limits (Figure 2).

The diagnosis of pemphigus vulgaris was based on the typical clinical and morphological criteria. Histopathological examination showed an intraepidermal acantholytic blister compatible with PV (Figure 1D). Direct immunofluorescence (DIF) displayed a weak intercellular deposition of IgG and C3 (Figure 1E). Indirect immunofluorescence (IIF) on a monkey esophagus substrate showed epithelial intercellular IgG with a 1:160 titer. Of note, Enzyme-linked immunosorbent assay (ELISA) was negative for both Dsg 1 and 3 (1.6 and 0.8 U/ml, respectively; negative, <14 U/ml; indeterminate, 14 to 20 for Dsg1 and negative < 7 U/ml; indeterminate, 7–20 for Dsg3) (MBL, Nagoya, Japan) instead, a 170 kDa band, obtained by IgG, was seen by immunoblotting (IB) with keratinocyte extracts (Figure 1F). The negative results on Dsgs were also confirmed by IB and different commercial Dsg1 and 3 ELISA kits (Euroimmun, Padova, Italia). Since epidermal growth factor receptor (EGFR) presents a molecular weight marker similar to the unknown antigen, we also performed an IB on keratinocyte extracts using an anti-EGFR antibody as control (Figure 1G). The results showed that the autoantigen did not comigrate with EGFR. The non-desmoglein autoantigen prompts us to term the case as atypical pemphigus vulgaris. The patient, after interrupting Imiquimod application, started treatment with prednisolone (80 mg iv/day) for 7 days, gradually tapered and finished after 4 weeks, with complete clearing of skin and mucosal lesions. The patient reported no physical or psychological sequelae, and he did not have any problem in returning to his everyday life. After reaching clinical remission without therapy, the patient underwent regular follow-up visits and after 2 years did not develop any relapse (Figure 2).

## Discussion

To the best of our knowledge, only four patients with pemphigus induced by Imiquimod have been reported. Two patients showed an intercellular IgG deposition by DIF, one was negative by DIF and one patient not tested (6–9). Three patients developed Imiquimod-induced erosions on the application site and only one DIF negative patient developed general bullae distant from the site of application (6). Circulating autoantibodies were tested by IIF in one patient who showed an intercellular staining with antibodies at a titer of 1:200 (7). To our knowledge no study investigated the reactivity to Dsg1 and 3 by ELISA and/or IB.

In the case reported herein the circulating autoantibodies failed to react against Dsg1 and Dsg3 while bound to an unknown keratinocyte antigen of 170 kDa. However, the anti-keratinocyte autoimmune response is demonstrated by intercellular labeling due to IgG deposition and circulating IgG which could provoke lesions distant from the site of application of Imiquimod. In this context, Imiquimod therapy has been reported to induce several autoimmune conditions such as psoriasis, vitiligo and exacerbations of myasthenia gravis (10).

A putative pemphigus autoantigen of 170 kDa could be EGFR. Several studies demonstrated that EGFR is involved in the cellular stress response that could lead to blister formation (2). Thus, its role in PV pathogenesis and the molecular weight similar to the unknown antigen prompt us to verify whether EGFR was an autoantibody target without success. Another possible autoantigen of 170 kDa could be alpha-2-macroglobulin-like-1 (A2ML1), a broad range protease inhibitor frequently targeted by paraneoplastic pemphigus autoantibodies. However, the reducing condition used in IB experiments did not allow to detect A2ML1 that is usually detected as native protein by immunoprecipitation assay (11). Thus, the 170 kDa antigen remains an unknown atypical pemphigus autoantigen and further studies are needed to characterize it.

Hypothetically, Imiquimod might induce pemphigus involving overproduction of IFN-alfa by dendritic cells and keratinocytes stimulated by TLR-7 with consequent induction and maintenance of autoreactive B-cells. However, it is worth mentioning that the increased levels of TNF-alfa, IL-1, IL-6, IL-10, and IL-12 have been detected in the serum of patients with pemphigus and increased serum levels of TNF-alfa, IL-1 and IL6 have been correlated with disease activity. In addition, anti–TNF-alfa was reported to be effective in the treatment of PV (12). Nevertheless, the development of PV 3 days following TLR7 stimulation suggests that the Imiquimod treatment unmasked subclinical disease. In fact, people who undergo Imiquimod therapy hardly ever develop pemphigus, suggesting that specific predisposing factors are needed. It could be hypothesized that the pre-existence of low titers of circulating autoantibodies in predisposed individuals, for example with specific human leukocyte antigen haplotype, together with the cytokine milieu induced by the Imiquimod therapy may favor pemphigus development.

A limitation of our study was the inability to characterize the 170 kDa antigen identified by IB on keratinocyte extracts. However, the strength of our study is the thorough immunological characterization of the patient, which is lacking in almost all the studies in literature.

## Conclusion

In some patients, topical application of imiquimod could induce pemphigus. Future studies could be carried out to assess the plausibility of the hypothesis that in the presence of multiple and/or voluminous BCC, sequential treatment with Imiquimod could reduce the risk of high absorption and adverse events.

## Data availability statement

The raw data supporting the conclusions of this article will be made available by the authors, without undue reservation.

## Ethics statement

Ethical review and approval was not required for the study on human participants in accordance with the local legislation and institutional requirements. The patients/participants provided their written informed consent to participate in this study. Written informed consent was obtained from the individual(s) for the publication of any potentially identifiable images or data included in this article.

## Author contributions

FrM and GD contributed to the conception and design of the study. DC, LF, and SP collected the clinical data and information. DC wrote the first draft of the manuscript. FeM, AS, and SR performed laboratory analysis, ELISA, and histology. FrM, GD, SR, and DC wrote sections of the manuscript. All authors contributed to the manuscript revision, read, and approved the submitted version.
